# HLA Associations in Classical Hodgkin Lymphoma: EBV Status Matters

**DOI:** 10.1371/journal.pone.0039986

**Published:** 2012-07-10

**Authors:** Xin Huang, Kushi Kushekhar, Ilja Nolte, Wierd Kooistra, Lydia Visser, Ilby Bouwman, Niels Kouprie, Rianne Veenstra, Gustaaf van Imhoff, Bianca Olver, Richard S. Houlston, Sibrand Poppema, Arjan Diepstra, Bouke Hepkema, Anke van den Berg

**Affiliations:** 1 Department of Pathology and Medical Biology, University of Groningen, University Medical Center Groningen, Groningen, The Netherlands; 2 Department of Pathology, Health Science Center, Peking University, Beijing, China; 3 Department of Epidemiology, University of Groningen, University Medical Center Groningen, Groningen, The Netherlands; 4 Department of Hematology, University of Groningen, University Medical Center Groningen, Groningen, The Netherlands; 5 Department of Laboratory Medicine, University of Groningen, University Medical Center Groningen, Groningen, The Netherlands; 6 Section of Cancer Genetics, Institute of Cancer Research, Sutton, United Kingdom; Karolinska Institutet, Sweden

## Abstract

The pathogenesis of classical Hodgkin lymphoma (cHL) involves environmental and genetic factors. To explore the role of the human leukocyte antigen (HLA) genes, we performed a case-control genotyping study in 338 Dutch cHL patients using a PCR-based sequence-specific oligonucleotide probe (SSOP) hybridization approach. The allele frequencies were compared to HLA typings of more than 6,000 controls. The age of the cHL patients varied between 13 and 81 years with a median of 35 years. Nodular sclerosis subtype was the most common subtype (87%) and EBV was detected in 25% of the cHL patients. HLA-B5 was significantly increased and HLA-DR7 significantly decreased in the total cHL patient population as compared to controls. Two class II associations were observed to be specific for the EBV− cHL population with an increase of HLA-DR2 and HLA-DR5. Allele frequencies of HLA-A1, HLA-B37 and HLA-DR10 were significantly increased in the EBV+ cHL population; these alleles are in strong linkage disequilibrium and form a common haplotype in Caucasians. The allele frequency of HLA-A2 was significantly decreased in the EBV+ cHL population. Analysis of haplotypes with a frequency of >1% revealed a significant increase of HLA-A2-B7-DR2 in EBV− cHL as compared to controls. SSOP association analysis revealed significant differences between EBV+ and EBV− cHL patients for 19 probes that discriminate between HLA-A*01 and HLA-A*02. In conclusion, the HLA-A1 and HLA-A2 antigens and not specific single nucleotide variants shared by multiple alleles are responsible for the association with EBV+ cHL. Furthermore several new protective and predisposing HLA class I and II associations for the EBV+, the EBV− and the entire cHL population were identified.

## Introduction

Classical Hodgkin lymphoma (cHL) is a typical multi-factorial disease with both environmental and genetic factors acting together to cause disease [1], [2]. Epidemiological studies reporting familial clustering of cHL [3] and racial variation in the incidence of cHL [4] give substantial support for an inherited risk to cHL. Genetic associations with specific Human Leukocyte Antigen (HLA) alleles have been reported in both sporadic and familial cHL [5]–[7].

Epstein Barr virus (EBV) is a well-established causal factor in a subset of cHL patients [8]. The expression pattern of EBV genes in Hodgkin Reed-Sternberg (HRS) cells is restricted to the two latent membrane proteins (LMP1 and LMP2) and the EBV nuclear antigen 1 (EBNA1) [8]. Despite the lack of immunodominant EBV proteins, LMP and EBNA1-specific T cell responses can be efficiently induced in the context of specific HLA class I or class II molecules [9]–[11]. The extreme diversity of HLA genes influences both the affinity and specificity of antigenic peptide binding and is responsible for variations in host anti-viral immune defenses. Genetic variation in host anti-viral immune responses related to HLA polymorphisms might be an important contributor to the development of virally induced malignancies.

Initial HLA association studies in cHL were performed without taking EBV status into account and associations of HLA-A1, HLA-B5, HLA-B8 and HLA-B18 with cHL have been described, although the degree of reproducibility was low [6], [12]–[14]. More recently, we focused on EBV stratified cHL subgroups in a genetic screening study of the entire HLA region [5]. In a subsequent finescreening analysis we found a strong association of specific HLA-A alleles with susceptibility to EBV+ cHL in Dutch and English patients [15]. HLA-A1 was associated with an increased risk for EBV+ cHL, whereas HLA-A2 was associated with a decreased risk for EBV+ cHL [16]. This association was confirmed in 934 Scandinavian and English cHL patients in a study by Hjalgrim et al [17]. In two recently performed genome wide association studies (GWAS) in cHL patients, the most significantly associated SNP (rs6903608) was located within the HLA class II region [18], [19].

In the present study, we performed an extensive screening of the HLA class I and II genes to investigate possible associations of HLA alleles in the total, the EBV+, and the EBV− cHL (sub)populations. Furthermore, we intended to establish whether specific single nucleotide polymorphism (SNP) positions in the HLA genes that might be shared by multiple HLA alleles are responsible for the observed genetic associations rather than specific HLA alleles.

## Materials and Methods

### Ethics statement

All patients gave written informed consent in accordance with the Declaration of Helsinki and the protocols were approved by the medical ethics board of the University Medical Center, Groningen.

### Patients and controls

183 cHL patients from the northern region of the Netherlands who participated in the previous population-based genotyping study [5] were included in the present study. 155 additional patients diagnosed and/or treated between 2000 and 2010 at the University Medical Center Groningen in the northern region of the Netherlands were also included. Data on gender, age, and histopathological diagnosis was available for all these patients. No data were available on family history. Classification according to the WHO was performed consistently with the previous study and cHL cases that could not be unequivocally subtyped (usually because of little tissue) were classified as cHL not otherwise specified (NOS) (Table 1). The presence of EBV in tumor cells was detected in formalin fixed paraffin embedded tissue sections by in-situ hybridization (ISH) with a fluorescin-conjugated PNA probe specific for the EBV-encoded EBER RNAs (DAKO, Glostrup, Denmark). The control group consisted of blood bank donors from the same geographical region typed for HLA-A (n = 7,554), HLA-B (n = 7,554) and HLA-DR (n = 6,559) by serological methods and in case of unclear results or apparent class II homozygosity additionally by DNA based methods.

**Table 1:** Distribution of sex, histological subtype and age in cHL population stratified by EBV status.

	Patients (n)	% EBV+
**Sex**		
Male	177	36%
Female	161	13%
**Histological subtype**		
NS	256	19%
MC	29	64%
LR	10	30%
NOS	43	31%
**Median age** (range)	35 (13–81)	

### HLA genotyping

Blood samples of cHL patients were collected and genomic DNA was extracted from the peripheral blood mononuclear cell pellets using standard laboratory protocols. The HLA genotype was analyzed at medium resolution by a polymerase chain reaction-based sequence-specific oligonucleotide probe hybridization (PCR-SSOP) approach using commercial kits (Gen-Probe, San Diego, CA) and Luminex xMAP technology (Luminex Corp., Austin, TX). The assays were performed according to the manufacturer's instruction in a European Federation for Immunogenetics accredited laboratory. Briefly, biotin-labeled amplification products were generated for exon 2 (for HLA-DRB, HLA-DPB1) and exon 3 (for HLA-A, HLA-B, HLA-Cw and HLA-DQB1) of the HLA genes, followed by a hybridization reaction with a series of SSOPs. Of the 401 PCR-SSOP probes 43 were either positive or negative for all samples and were therefore excluded from the analyses. Of the 358 included probes, 63 were for the HLA-A locus, 83 for the HLA-B locus, 53 for the HLA-C locus, 72 for the HLA-DRB locus, 41 for the HLA-DQB1 locus and 46 for the HLA-DPB1 locus. Each probe covered 1 to 3 SNP positions. The HLA alleles were defined by specific hybridization patterns of multiple probes. All analyses were restricted to the broad alleles because of missing split allele data for a substantial proportion of the control population. HLA genotype was ascertained according to the manufacturers instructions using the manufacturer's software and additionally by the SCORE software [20], enabling the exclusion of probes with borderline hybridization signals. The presumed antigen or T cell receptor binding function of the SNPs were identified according to Bjorkman and Parham [21].

HLA allele frequencies of the cHL patients were deducted from the PCR-SSOP genotyping data based on the Nomenclature for factors of the HLA system (http://hla.alleles.org/nomenclature/naming.html). In case of ambiguous results, the allele combination only consisting of common and well documented alleles [22] were used. For HLA-DPB1 one ambiguity remained (DPB1*04:02/105:01) which was analyzed as one group of alleles.

### Statistical analysis

For each individual PCR-SSOP association with EBV status was tested by logistic regression in PLINK v1.07 [23] (http://pngu.mgh.harvard.edu/purcell/plink/) with EBV status as a dependent variable, SSOP as an independent variable, and age and histopathological diagnosis of cHL as confounding covariates. Linkage disequilibrium (LD) between the SSOPs was determined using the measures D' and r^2^. The first measure reflects the evolutionary history of the SSOP pairs in which a D' of 1 implies no recombination between the two SSOPs. The latter measure is the correlation between SSOPs and reflects if SSOP associations are independent of each other (higher r^2^ means less independence).

Allele frequencies of HLA-A, HLA-B and HLA-DR of the total cHL group, the EBV+ subgroup and the EBV− subgroup were compared with allele frequencies of the controls and significant differences were assessed by Chi-square tests and odds ratio (OR) to quantify the difference and estimate risk were determined. Allele frequencies for HLA-C, HLA-DP and HLA-DQ of the controls were not available. All HLA genes were included for the analysis between EBV+ and EBV− cHL patients. Alleles with a frequency <1% in our population were excluded for all these analyses.

Haplotype frequencies for HLA-A-B-DR were estimated in the controls, the cHL patients, the EBV+ and the EBV− patients using PHASE v2.1^24,25^. For the comparison between EBV+ and the EBV− patients we analyzed haplotype frequencies for HLA-A-B-C-DR-DQ-DP and for HLA-A-B-C-DR-DQ without DP because of the known recombination hotspot between DQ and DP. Next the estimated counts (calculated by sample size x estimated frequencies) of each haplotype were compared between cHL, EBV+ and EBV− patients and controls and between EBV+ and EBV− patients using a chi-square test and the OR and 99.9% CI were determined. Only those haplotypes with a frequency of >1% (in patients plus controls) were considered to be relevant in the analysis.

Our patient cohort was used in the replication series of the recently published GWAS that identified the HLA class II SNP rs6903608 as a highly significant susceptibility marker for cHL [18,18]. We now compared the SNP alleles (C and T) with the HLA typing data to investigate their linkage disequilibrium (LD). Combined HLA typing and SNP data were available for 278 cHL patients. Two-marker haplotypes of the SNP rs6903608 and the HLA typing were constructed per gene using PHASE v2.1 [24], [25]. Frequencies of HLA typing among haplotypes with a C allele and a T allele at rs6903608 were compared per HLA typing using the Chi-square test.

For each phenotype and PCR-SSOP a test was performed, hence a correction for multiple testing was required. Because LD exists between the PCR-SSOPs and between the HLA phenotypes, and because the HLA-phenotypes are derived from the PCR-SSOPs, a Bonferroni test for 401 PCR-SSOPs and 74 HLA phenotypes would be too conservative. Therefore, we considered p-values smaller than 0.001 to be significant at a level of 5% and p-values <0.003 to be suggestive for association. Further more 99.9% confidence intervals for the Ors were determined.

## Results

### Clinicopathological characteristics

Characteristics of the patient population in terms of age, gender and histopathological subtype in relation to EBV status is summarized in Table . EBV was present in the tumor cells in 78 (25%) of the 311 cHL patients (for 27 patients EBV status was unknown). In the total group, median age at diagnosis was 35 years (range 13 to 81) and the percentage of males was 52%. Age at time of diagnosis was similar in the EBV+ and EBV− group, i.e. 37 (range 17 to 70) in EBV+ and 32 (range 13 to 81) in EBV−. 43 of 338 cHL patients could not be subtyped and were designated cHL NOS. In the remaining patients, nodular sclerosis (NS) was the most common subtype accounting for 87%. Mixed cellularity (MC) and lymphocyte rich (LR) subtypes were less common with frequencies of 10% and 3%, respectively. The lymphocyte depletion (LD) subtype was absent in this patient group. These characteristics are largely consistent with those published for Caucasian cHL populations.

### Allele frequency differences compared to controls

An overview of all allele frequency data, OR and p-values is given in Supplementary Table S1. HLA allele frequencies in the control population were consistent with previously published data [26]. HLA phenotype frequencies including OR and p-values are given in Supplementary Tables S2, S3, S4 and Supplementary Figure S1. This paper focuses on the HLA allele frequency analysis. Overall, the significant associations observed in the allele frequency analysis were similar to the phenotype frequency analysis, with some minor differences only in the overall cHL population.

**Figure 1:**
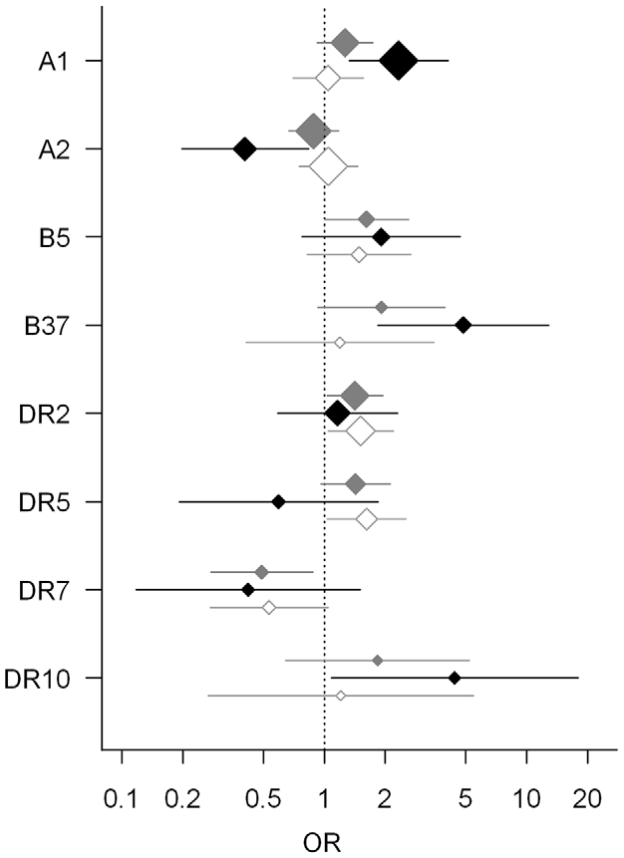
Odds ratios and 99.9% confidence intervals of the genotype allele frequencies. The graph shows the (nearly) significant differences between the controls and either the total cHL patient group (grey), the EBV+ (black), or the EBV− (white) subgroup of patients. The size of the diamonds reflects the allele frequency.

In the cHL patient group the allele frequencies of HLA-B5 and HLA-DR2 were significantly increased as compared to the controls (8.3% vs 5.3%; p = 8.9x10^−4^, 21.7% vs 16.4; p = 3.1×10^−4^). The HLA-DR7 allele frequency was significantly decreased (5.2% vs 10.1%; p = 3.7×10^−5^) (Table 2; Figure 1). In EBV− cHL patients a significantly increased frequency was observed for HLA-DR2 and HLA-DR5 compared to controls (22.9% vs 16.4%; p = 2.1×10^−4^, 14.3% vs 9.3%; p = 3.6×10^−4^) (Table 3, Figure 1). For the EBV+ cHL subgroup strong associations were observed for HLA-A1, HLA-A2, HLA-B37 and HLA-DR10 (Table 3; Figure 1). In contrast to the significant increased frequency of HLA-DR5 in the EBV− cHL subgroup, the frequency was reduced in the EBV+ cHL subgroup, although not significant. The HLA-A1 frequency was increased compared to the controls (33.3% vs 17.7%; p = 3.5×10^−7^), whereas the HLA-A2 frequency was decreased (16.0% vs 32.0%; p = 2.1×10^−5^) in EBV+ cHL. For HLA-B37 (8.3% vs 1.8%; p = 3.3×10^−9^) and HLA-DR10 (3.8% vs 0.9%; p = 1.4×10^−4^) an increased frequency was observed in the EBV+ cHL patients as compared to the controls.

**Table 2:** HLA allele frequencies of HLA-A, HLA-B, and HLA-DR alleles with a (nearly) significant difference between controls and cHL patients.

	Controls		cHL patients		Controls vs cHL
	n	%	n	%	(*P-value* *)
HLA-B5	798	5.3	55	8.3	**8.9×10^−4^**
HLA-B37	277	1.8	23	3.5	*2.7×10^−3^*
HLA-DR2	2152	16.4	146	21.7	**3.1×10^−4^**
HLA-DR5	1224	9.3	86	12.8	*2.8×10^−3^*
HLA-DR7	1320	10.1	35	5.2	**3.7×10^−−5^**

* Significant differences (p<0.001) are shown in bold, suggestive ones (p<0.003) in italic.

**Table 3:** Allele frequencies of HLA-A, HLA-B, and HLA-DR alleles with (nearly) significant difference between controls and EBV+ or EBV− cHL subgroups.

	Controls		EBV+ cHL		EBV− cHL		Controls vs EBV+	Controls vs EBV−
	n	%	n	%	n	%	(P-value*)	(P-value*)
HLA-A1	2667	17.7	52	33.3	85	18.3	**3.5×10^−7^**	NS
HLA-A2	4828	32.0	25	16.0	153	33.0	**2.1×10^−5^**	NS
HLA-B5+	798	5.3	15	9.6	35	7.6	NS	NS
HLA-B37	277	1.8	13	8.3	10	2.2	**3.3×10^−9^**	NS
HLA-DR2	2152	16.4	29	18.6	106	22.9	NS	**2.1×10^−4^**
HLA-DR5	1224	9.3	9	5.8	66	14.3	NS	**3.6×10^−4^**
HLA-DR7	1320	10.1	7	4.5	26	5.6	NS	*1.7×10^−3^*
HLA-DR10	118	0.9	6	3.8	5	1.1	**1.4×10^−4^**	NS

+ HLA-B5 is included because these alleles had significantly different frequencies in the total group of cHL cases compared with controls (Table 2). *Significant differences (p<0.001) are shown in bold, suggestive ones (p<0.003) in italic.

### Differences in HLA allele frequencies between EBV+ and EBV− cHL patients

Comparison of the HLA allele frequencies revealed four significant differences between the EBV+ and EBV− cHL subgroups (Supplementary Table S1). The HLA-A1 frequency was significantly increased (33.3% vs 18.3%; p = 9.2×10^−5^) and the HLA-A2 frequency was significantly decreased (16.0% vs 33.0%; p = 5.2×10^−5^). In addition, we observed a significantly increased frequency for HLA-B37 (8.3% vs 2.2%; p = 4.8×10^−4^) (Table 4).

**Table 4:** HLA allele frequencies with a (nearly) significant difference between EBV+ and EBV− cHL patients.

	EBV+ cHL		EBV− cHL		p-value*
	n	%	n	%	
HLA-A1	52	33.3	85	18.3	**9.2×10^−5^**
HLA-A2	25	16.0	153	33.0	**5.2×10^−5^**
HLA-B37	13	8.3	10	2.2	**4.8×10^−4^**
HLA-Cw6	21	13.6	27	5.9	*2.2×10^−3^*

* Significant differences (p<0.001) are shown in bold, suggestive ones (p<0.003) in italic. HLA-Cw6 is included because it is part of a common haplotypen in the Caucasian population.

### Haplotype analysis

Haplotype frequency data are shown in Supplementary Table S5. For the comparison of the total cHL population versus controls no significant associations were observed for haplotypes with a frequency of >1%. Two suggestive differences were observed, i.e. A2-B7-DR2 (3.2% vs 5.3%, p = 2.1×10^−3^) and A2-B40-DR6 (2.1% vs 0.4%, p = 1.9×10^−3^). The A2-B7-DR2 haplotype was significantly increased in controls vs EBV− cHL, i.e. 3.2% vs 6.7% (p = 2.2×10–5), whereas none of the haplotypes showed a significant difference between EBV+ cHL and controls. Overall, the p-values for the individual HLA alleles revealed much stronger associations as compared to the associations observed for the haplotype analysis.

### Association analysis of individual PCR-SSOPs in EBV+ and EBV− cHL patients

**Figure 2:**
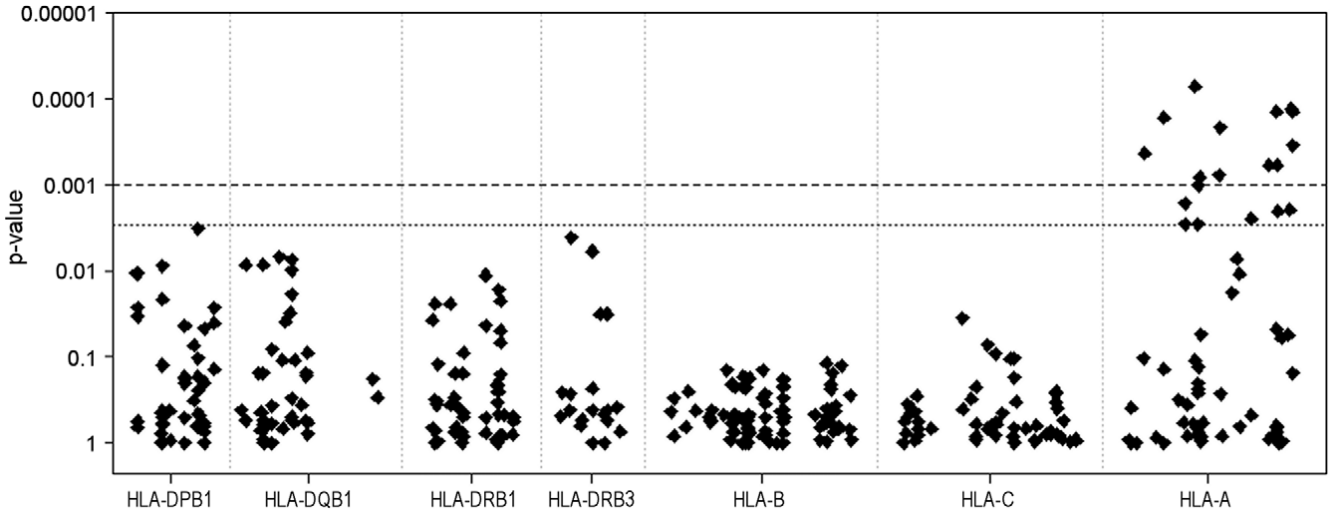
Genetic association of individual PCR-SSOP in EBV+ cHL. The p-value of each SSOP for differences in frequencies between EBV+ and EBV− cHL cases is plotted on the y-axis. Genes are ordered according to their relative positions on the short arm of chromosome 6 (6p-telomere to 6p-centromere). Strong associations with EBV status were present only for part of the PCR-SSOPs within the *HLA-A* gene.

**Figure 3:**
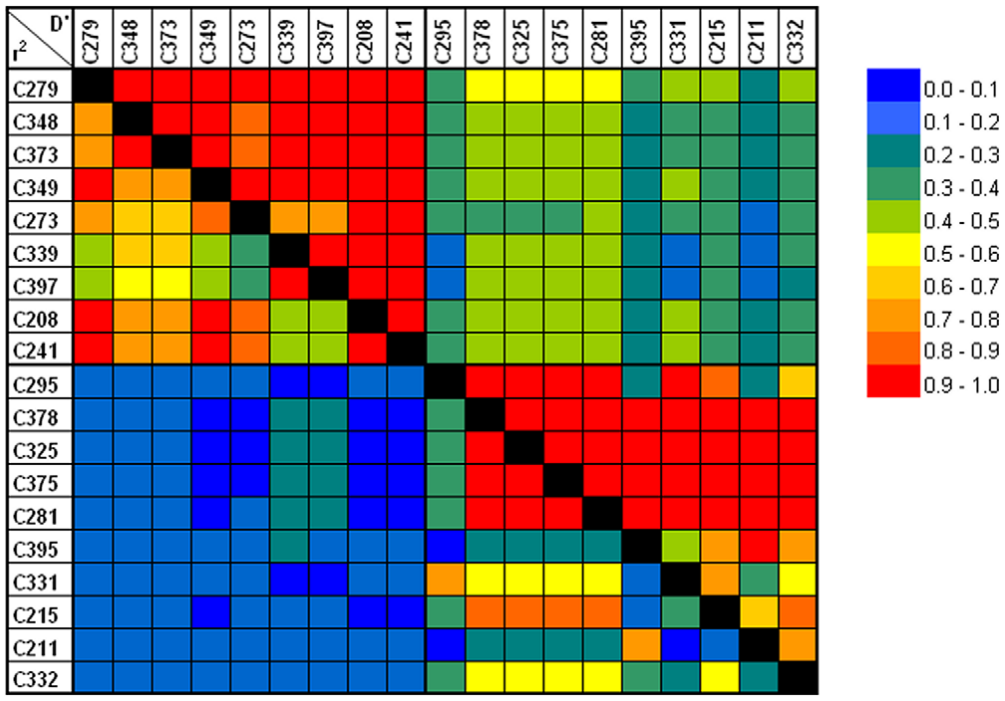
LD between the associated HLA-A SSOPs. Below the diagonal the r^2^ values are given and above the diagonal the D' values. The SNPs are sorted according the HLA-A allele specificity and strength of association with EBV status (same order as table 5). SSOPs C279–C241 in the left/upper part are specific for HLA-A*02 and SSOPs C295–C332 in the right/lower are specific for HLA-A*01.

Analysis of each of the 358 PCR-SSOPs revealed a significant difference between EBV− and EBV+ cHL patients for 19 HLA-A gene PCR-SSOPs (Figure 2; Supplemental Table S1). Ten of the PCR-SSOPs were specific for the HLA-A*01 allele and were more common in EBV+ cHL patients, while the other nine PCR-SSOPs specific for the HLA-A*02 allele were less common in this patient group (Table 2). Interestingly the most significant SSOP (C295) is not strongly correlated with any of the other SSOPs (r^2^<0.4 except for pair C295-C331 [r^2^ = 0.70]) (Figure 3). This implies that the association of C331 is likely caused by LD with C295. These probes share a specificity for HLA-A*23/*A24 next to HLA-A*01. In addition, we observed a second independent association for the SSOPs C395 and C211, which were the only two probes that shared specificity for HLA-A*03. A third independent association was shown for the SSOPs C378, C325, C375, C281 and C215. Specificity of these SSOPs is largely restricted to HLA-A*01. The C332 SSOP showed an intermediate association. The HLA-A*02 specific SSOPs are strongly correlated with each other (most pairs have r^2^>0.8, for all pairs r^2^>0.3) and hence there is likely only one signal that is causing these associations. No LD is observed between the HLA-A*01 and the HLA-A*02 specific SNPs (r^2^<0.23; D'<0.53).

The 19 SSOPs with significant differences contained polymorphic residues within nine of the HLA-A*02-specific and ten of the HLA-A*01 specific SSOPs that are located at key positions in the peptide binding pockets of the HLA molecules (Table 5). PCR-SSOPs that were less specific for the HLA-A*01 or HLA-A*02 alleles showed a similar trend in the OR as the significant PCR-SSOPs but with lower p-values (Supplementary Table S1).

**Table 5:** Overview of PCR-SSOPs with significant differences between EBV+ and EBV− cHL population.

SSOP	Specificity of probe^a^	AA positions (IMGT)	Potential contact position^†^	EBV+ (%)	EBV− (%)	P	OR
c279	A*02	9–12,23–25	Peptide (9,24)	23.0	50.0	**4.3×10^−4^**	0.32
c348	A*02, A*68, A*69	141–142, 144–146	Peptide (146), TCR (145,146)	34.6	60.3	**6.0×10^−4^**	0.37
c373	A*02, A*68, A*69	143–146, 150–153	Peptide (143,152), TCR (145,146,150,151)	34.6	60.3	**6.0×10^−4^**	0.37
c349	A*02	94–98	Peptide (95,97)	26.9	53.0	**7.6×10^−4^**	0.36
c273	A*02, A*31, A*33, A*74	69–72, 76–79	Peptide (70,77), TCR (69,72,76,79)	31.6	58.2	**8.1×10^−4^**	0.37
c339	A*02, A*24, A*68, A*69	150–154	Peptide (152), TCR (150,151,154)	48.7	71.6	*2.0*×*10^−3^*	0.41
c397	A*02, A*23, A*24, A*68, A*69	124–128		51.3	72.8	*2.5*×*10* ^−*3*^	0.41
c208	A*02	60–65	Peptide (62,63), TCR (61,62,65)	29.5	53.0	*2.9*×*10* ^−*3*^	0.41
c241	A*02	70–71, 73–74, 76	Peptide (70,73 74), TCR (76)	29.5	53.0	*2.9*×*10^−3^*	0.41
c295	A*01, A*23, A*24, A*25, A*26, A*30, A*32, A*36, A*74	70–74	Peptide (70,73,74), TCR (72)	80.8	53.9	**7.2×10^−5^**	3.79
c378	A*01	156, 158, 166–168	Peptide (156,167), TCR (158,166,167)	56.4	29.9	**1.3×10^−4^**	3.05
c325	A*01, A*36	150–152	Peptide (152)TCR (150,151)	56.4	30.2	**1.4×10^−4^**	3.03
c375	A*01	161–163, 166–168	Peptide (163,167), TCR (162,163,166,167)	56.4	30.2	**1.4×10^−4^**	3.03
c281	A*01, A*36	41–44		55.8	29.3	**1.7×10^−4^**	3.02
c395	A*01, A*03, A*11, A*30, A*36	93–98	Peptide (95,97)	84.6	61.2	**2.1×10^−4^**	3.79
c331	A*01, A*23, A*24	164, 166–168	Peptide (167), TCR (166,167)	71.8	47.0	**3.4×10^−4^**	2.96
c215	A*01, A*26, A*29, A*36	74–77	Peptide (74,77), TCR (76)	59.0	35.5	*1.0*×*10* ^−*3*^	2.58
c211	A*01, A*03, A*11, A*30, A*31, A*32, A*36, A*74	59–60, 62–64	Peptide (59,62,63), TCR (62)	87.2	68.0	*1.6*×*10* ^−*3*^	3.36
c332	A*01, A*11, A*25, A*26	160–163, 165	Peptide (163), TCR (162,163)	66.7	45.9	*2.0*×*10* ^−*3*^	2.49

a Only CWD alleles were included; for complete probe reactivity see http://www.gen-probe.com/; ^†^Contact position according to Bjorkman 1990.^18^. *Significant differences (p<0.001) are shown in bold, suggestive ones (p<0.003) in italic.

Analysis of the NS and the non-NS subgroups separately for the most significant PCR-SSOPs revealed trends similar to the total group albeit with lower p-values because of the smaller group sizes. Remarkably, the ORs in the non-NS group were more pronounced (0.21–0.23 for the HLA-A*02 SSOPs and 4.74–8.06 for HLA-A*01 SSOPs) than the ORs in the NS subgroup (0.36–0.44 for HLA-A*02 SSOPs and 2.31–3.49 for the HLA-A*01 SSOPs). Nevertheless, there was no significant difference between the NS and the non-NS subgroups for these SSOPs (p-values for interaction effects of SSOP by subtype on EBV status were all >0.05).

### LD of rs6903608 with HLA alleles

LD analysis of rs6903608 with the HLA typing revealed 15 significant associations (Table 6). Three of these associations were in agreement with the HLA allele frequency results, i.e. HLA-DR2, HLA-DR5 and HLA-DR7. The T allele was significantly associated with HLA-DR7 (8.6% vs 0.2%, p = 5.9×10^−6^). The C allele was significantly associated with HLA-DR2 and HLA-DR5 (49% vs 0.5%, p<2×10^−16^ and 23% vs 4%, p = 4.6×10^−12^). Eleven of the SNP / HLA allele associations, i.e. HLA-B7, HLA-B8, HLA-C7, HLA-DR1, HLA-DR3, HLA-DR4, HLA-DR8, DQB1, DQB2 and DQB4 were not significantly associated with cHL in the HLA allele frequency analysis in this study. However, HLA-DR4 was significantly associated with the total cHL group in the phenotype analysis (see Supplementary data).

**Table 6:** Association of the rs6903608 SNP alleles with HLA alleles in 278 cHL patients.

HLA allele		C-allele*	T-allele*	p-value
**HLA-A**		**n = 244**	**n = 310**	
1		13.4%	28.2%	2.7×10^−5^
**HLA-B**		**n = 241**	**n = 307**	
7		34.2%	6.7%	4.4×10^−16^
8		8.1%	21.4%	2.0×10^−5^
**HLA-Cw**		**n = 237**	**n = 301**	
7		51.6%	3.41%	2.1×10^−6^
**HLA-DR**		**n = 244**	**n = 310**	
1		0.5%	17.6%	3.1×10^−11^
2		48.5%	0.5%	<2×10^−16^
3		4.3%	21.2%	9.2×10^−9^
4		0.5%	22.2%	2.2×10^−14^
5		22.9%	3.6%	4.6×10^−12^
7		0.2%	8.6%	5.9×10^−6^
8		0%	5.8%	1.3×10^−4^
**HLA-DQ**		**n = 243**	**n = 307**	
1		71.7%	36.1%	<2×10^−16^
2		5.0%	25.0%	2.4×10^−10^
4		0%	5.2%	3.4×10^−4^
**HLA-DP**		**n = 244**	**n = 310**	
1		0.8%	6.8%	1.3×10^−4^

* Indicated are the number of alleles (the total number of alleles is 556).

## Discussion

Inherited variations within the HLA genes as well as variations in host immune responses have long been recognized to be associated with susceptibility to disease, including cHL [27]. A number of HLA genes, alleles and serotypes have previously been reported to be involved in the pathogenesis of cHL [5]–[7] and GWAS indicated the most significant associations for cHL to be within the HLA locus [18], [19]. In this study we observed multiple associations that are specific for the EBV+, the EBV− or the total cHL subpopulations. HLA-B5 is a risk allele for cHL and HLA-DR7 is a protective allele irrespective of the EBV status. HLA-DR2 and HLA-DR5 are associated with an increased susceptibility in the EBV− cHL groups. In the EBV+ cHL population an increased susceptibility was observed for HLA-A1, HLA-B37 and HLA-DR10, whereas resistance to disease development was observed for HLA-A2.

Two GWAS indicated that rs6903608 was strongly associated with the entire cHL population and the EBV− cHL subpopulation respectively [18], [19]. To explore associations of the T and C alleles with certain HLA alleles, we compared the SNP data of our cohort to the HLA typing data and observed a significant LD with 15 HLA alleles (Table 6). Three of the 15 HLA alleles that were in strong LD with rs6903609, i.e. HLA-DR2, HLA-DR5 and HLA-DR7 were significantly associated with cHL in both the HLA allele and phenotype frequency analysis. HLA-DR4 was only significant in the phenotype analysis. The C-allele was frequently observed together with HLA-DR2 or HLA-DR5, i.e. 71.4% (48.5%+22.9%), while either HLA-DR allele was observed only in 4.1% in combination with the T-allele. These two HLA alleles were associated specifically with the EBV− cHL subgroup in our study. Since most cHL cases are EBV−, this association might explain the SNP association seen in the total cHL irrespective of EBV. Because the SNP was associated with many HLA alleles that were themselves not associated with cHL, the strong association of the SNP with cHL is probably caused by its strong LD with the HLA-DR alleles and not vice versa. Thus, by applying HLA typing we were able to identify the HLA genes and alleles that underlie the strong genetic association identified in a recent genome wide association study (GWAS) [18], [19].

The previously reported association of the HLA-A gene with EBV+ cHL [5], [15], [16] was confirmed both in comparison to EBV− cHL and in comparison to healthy controls with an increased risk for HLA-A*01 and a reduced risk for HLA-A*02. In contrast to the Caucasian population, we identified a protective effect for HLA−*02:07 for EBV− cHL and a predisposing effect for HLA-A*02:07 carriers for EBV+ cHL in the Chinese population [28]. The HLA-A*02(non02:07) typings showed no significant associations with either EBV stratified cHL. A tentative explanation for the HLA-A*02 associations are the known cytotoxic T cell responses to LMP1, LMP2 and lytic EBV derived antigenic peptides presented in the context of most HLA-A*02 [9], [29], [30] types, with the exception of HLA-A*02:07. In contrast no HLA-A*01 restricted cytotoxic EBV peptides have been found [31].

Analysis of the individual PCR-SSOPs revealed a significant difference for 19 out of the 358 probes in EBV+ as compared to EBV− cHL. These probes discriminated between the HLA-A*01 and HLA-A*02 alleles and showed only a limited number of cross reacting other alleles. The other, less significant HLA-A*01 and HLA-A*02 identifying probes showed a similar risk pattern. The lower significance level of these probes can be explained by their broader specificities including some common HLA-A alleles. For example, several probes share specificity for both HLA-A1 and HLA-A11. HLA-A*11 was previously reported to be a protective allele in EBV-associated undifferentiated nasopharyngeal carcinoma (UNPC) in both endemic and non-endemic regions [32], [33]. UNPC has the same EBV latency pattern as cHL with expression of only LMPs and EBNA1 and it was suggested that HLA-A*11 can efficiently present antigenic peptides from these proteins [29], [30]. Therefore, specificity of a probe for both HLA-A*11 and HLA-A*01 might diminish the strength of its association with EBV+ cHL. In combination with the LD analysis, we now showed that there is no evidence that individual SNPs detected by the PCR-SSOPs are important for the HLA-A*02 association, indicating that the complete HLA-A*02 allele is important for this association. For HLA-A*01 three clear independent signals were observed, representing different allele specificities in addition to the HLA-A*01 allele, i.e. HLA-A*23/*34 for C295 and C331, HLA-A*03 for C395 and C211, and no additional specificities for two of the five SSOPs of the third signal. The frequencies of the shared HLA-A alleles were similar in EBV− and EBV+ cHL cases indicating that the HLA-A*01 specificity is the most likely explanation for the observed associations.

The strong LD observed in the HLA region might affect the results of our HLA association studies. The HLA-A1 antigen is known to be in strong LD with HLA-B8 and in contrast to HLA-A1, HLA-B8 is capable of presenting EBV-derived peptides [34]. We observed that in the EBV+ cHL population 53% (23/43) of the HLA-A1 positive patients also carried the HLA-B8 allele, whereas in the EBV− cHL population this percentage was 66% (46/70). In theory, presence of HLA-B8 in HLA-A1 carrying EBV+ cHL might overcome the less effective presentation of EBV derived peptides by HLA-A1. Despite this known LD we found a very significant effect for HLA-A1 in this study consistent with previous studies [5], [15], [16] and no significant differences for HLA-B8. Haplotype analysis for A1-B8 also indicated no significant differences. The capacity of HLA-B8 to present EBV-derived peptides and the strong LD of HLA-B8 with HLA-A1 cannot explain the observed association in EBV+ cHL cases with HLA-A1.

The predisposing effects of HLA-B37 and HLA-DR10 for EBV+ cHL might at least be partly attributed to their strong LD with HLA-A1 since HLA-A1-B37-Cw6-DR10-DQ5 is a rather common haplotype in the Caucasian population. In our study, 12 of 13 HLA-B37+ and five of six HLA-DR10+ EBV+ cHL patients also carried HLA-A1. Haplotype analysis confirmed a significant increase in the A1-B37-DR10 haplotype frequency in EBV+ cHL (3.1%) as compared to controls 0.2% (p = 1.6×10^−12^). It should be noted that this haplotype is only observed in a small proportion of the HLA-A1 carriers and that despite the low p-value can not account for the observed significant associations of HLA-A1 with EBV+ cHL.

Two associations (HLA-B5 and HLA-DR7) were specific for the total cHL subgroup irrespective of the EBV status. The HLA-B5 association has been reported previously [35]. Significant effects of HLA-A1, HLA-B8, HLA-B18 and HLA-DR5 for cHL that have been reported in previous publications [12]–[14], [36] could not be confirmed in the total patient group in our study. These differences might be explained by differences in sample size, patient selection, and proportion of EBV+ cases, since we found HLA-A1 specifically in EBV+ cHL an HLA-DR5 specifically in EBV− cHL. Moreover, previous studies were carried out in relatively small numbers of patients ranging from 11 to 137 within different countries such as Egypt, Czech Republic, Sweden, Denmark, and United States. Distribution of HLA alleles varies widely by ethnicity and geography, which might lead to differences in disease-associated HLA alleles among different ethnic groups and geographic locations. A limitation of this approach is the inability to discriminate between specific allele variants, which might be more or less common in different populations.

The HLA-DR2 and HLA-DR5 class II associations were specific for the EBV− cHL subgroup. For HLA-DR5 we found a non-significant opposite effect in EBV+ cHL. An association with DRB1*15:01 has been reported previously to be associated with familial NS subtype cHL [7]. HLA-DR2 is a broad allele that includes the HLA-DR15 split allele and HLA-DR15 is the serological name for the HLA-DRB1*15:01 typing. This previously reported association is thus similar to the association that we found to be specific for EBV− cHL.

The HLA-A1 and HLA-A2 class I associations were specific for the EBV+ cHL subgroup, whereas the HLA class II associations were specific for the EBV− cHL subgroup. HLA class II molecules present exogenous antigenic peptides to CD4+ helper T cells, whereas HLA class I molecules bind and present peptides derived from endogenous proteins to CD8+ cytotoxic T lymphocytes [27]. Our findings thus support a differential immunogenic basis with a more prominent role in the effector phase of the immune response for HLA class I in EBV+ cHL and an immunoregulatory role for HLA class II in the pathogenesis of EBV− cHL. http://bloodjournal.hematologylibrary.org/cgi/content/full/92/10/3515 – B31This suggests presence of a specific HLA class II restricted antigen involved in the pathogenesis of EBV− cHL. Whether this is a pathogen-derived antigen or an antigen derived from a mutated protein remains unknown.

In conclusion, the current study confirms the previously reported genetic association of the HLA-A1 and HLA-A2 in EBV+ cHL. The genetic influence of these two HLA antigens is more important than individual SNPs that might be shared by multiple HLA-A alleles. In addition, we demonstrated two significant associations (HLA-B5 and HLA-DR7) for the cHL population and two significant HLA class II associations (HLA-DR2 and HLA-DR5) specific for the EBV− cHL population. The two new associations for the EBV+ cHL subgroup (HLA-B37 and HLA-DR10) are most likely related to the common haplotype including HLA-A1. Overall, the haplotype analyses did not show very strong associations as compared to analysis of the individual genes. This indicates that the individual genes are responsible for the associations between cHL and the HLA region. Our study implies an influence of the interaction between environmental and genetic risk factors in the development of cHL and supports heterogeneity in the genetic predisposition to EBV+ and EBV− cHL.

## Supporting Information

Figure S1 **Odds ratios and 99.9% confidence intervals of the phenotype allele frequencies.** It shows the (nearly) significant differences between the blood bank controls and either the total cHL patient group (grey), the EBV+ (black), or the EBV− (white) subgroup of patients. The size of the diamond reflects the allele frequency.
(TIFF)

Table S1 **Genotype and phenotype analysis for all broad HLA alleles and SSOP analysis.**
(XLS)

Table S2 **Phenotype frequencies of HLA-A, HLA-B, and HLA-DR alleles with a (nearly) significant difference between blood bank controls and cHL patients.**
(DOC)

Table S3 **Phenotype frequencies of HLA-A, HLA-B, and HLA-DR alleles with (nearly) significant difference between blood bank controls and EBV+ cHL subgroups and between controls and EBV− cHL.**
(DOC)

Table S4 **Phenotype frequencies of HLA alleles with (nearly) significant difference between EBV+ or EBV− cHL patients.**
(DOC)

Table S5 **Haplotype association analyses for the HLA genes.**
(XLS)
