# Supplementary figures and images for: HLA Associations in Classical Hodgkin Lymphoma: EBV Status Matters
Source: PLoS One. 2012 Jul 10;7(7):e39986. doi: 10.1371/journal.pone.0039986 (PMC3393726; doi:10.1371/journal.pone.0039986)

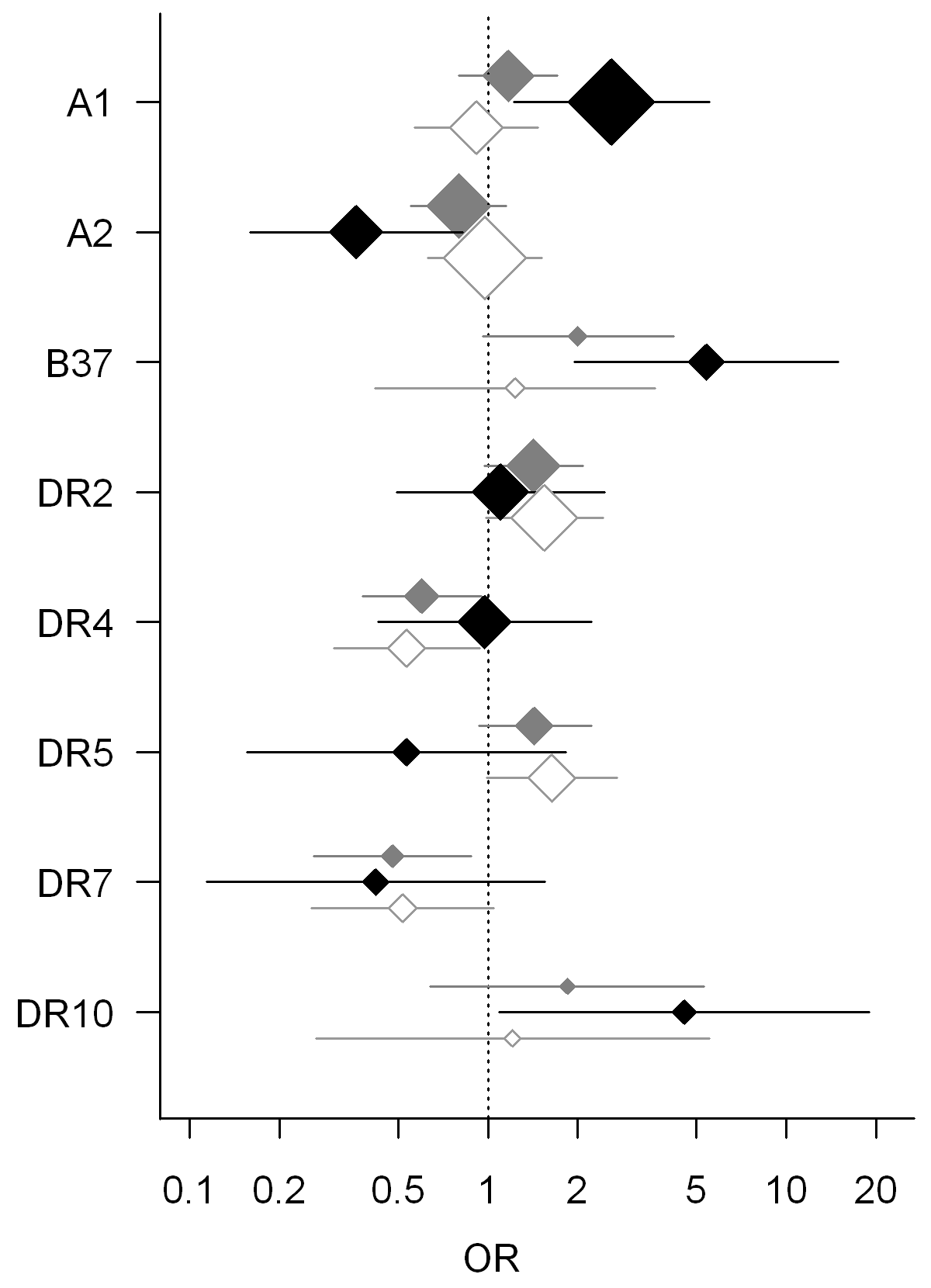

Supplement: Figure S1 — Odds ratios and 99.9% confidence intervals of the phenotype allele frequencies. It shows the (nearly) significant differences between the blood bank controls and either the total cHL patient group (grey), the EBV+ (black), or the EBV− (white) subgroup of patients. The size of the diamond reflects the allele frequency. (TIFF) [file pone.0039986.s001.tiff]
