# Supplementary material for: HLA Associations in Classical Hodgkin Lymphoma: EBV Status Matters
Source: PLoS One. 2012 Jul 10;7(7):e39986. doi: 10.1371/journal.pone.0039986 (PMC3393726; doi:10.1371/journal.pone.0039986)
Supplement: Table S2 — Phenotype frequencies of HLA-A, HLA-B, and HLA-DR alleles with a (nearly) significant difference between blood bank controls and cHL patients. (DOC) [file pone.0039986.s003.doc]

**Supplementary Table S2.** Phenotype frequencies of HLA-A, HLA-B, and HLA-DR alleles with a (nearly) significant difference between blood bank controls and cHL patients

| **Allele** | **Controls** | |  | **cHL patients** | |  | **Controls vs cHL** |
| --- | --- | --- | --- | --- | --- | --- | --- |
|  | n | % |  | n | % |  | p ***** |
| HLA-B37 | 270 | 3.6 |  | 23 | 6.9 |  | *1.6x10^-3^* |
| HLA-DR2 | 1967 | 30.0 |  | 127 | 37.8 |  | *2.4x10^-3^* |
| HLA-DR4 | 1969 | 30.0 |  | 69 | 20.5 |  | **2.0x10^-4^** |
| HLA-DR7 | 1246 | 19.0 |  | 34 | 10.1 |  | **4.5x10^-5^** |

*****Significant differences (p<0.001) are shown in bold, suggestive ones (p<0.003) in italic.
