# Supplementary material for: HLA Associations in Classical Hodgkin Lymphoma: EBV Status Matters
Source: PLoS One. 2012 Jul 10;7(7):e39986. doi: 10.1371/journal.pone.0039986 (PMC3393726; doi:10.1371/journal.pone.0039986)
Supplement: Table S3 — Phenotype frequencies of HLA-A, HLA-B, and HLA-DR alleles with (nearly) significant difference between blood bank controls and EBV+ cHL subgroups and between controls and EBV− cHL. (DOC) [file pone.0039986.s004.doc]

**Supplementary Table S3.** Phenotype frequencies of HLA-A, HLA-B, and HLA-DR alleles with (nearly) significant difference between blood bank controls and EBV+ or EBV- cHL subgroups

| **Allele** | **Controls** | |  | **EBV+ cHL** | |  | **EBV- cHL** | |  | **Controls vs EBV+** | **Controls vs EBV-** |
| --- | --- | --- | --- | --- | --- | --- | --- | --- | --- | --- | --- |
|  | n | % |  | n | % |  | n | % |  | p ***** | p ***** |
| HLA-A1 | 2426 | 32.1 |  | 43 | 55.1 |  | 70 | 30.2 |  | **1.5x10^-5^** | ns |
| HLA-A2 | 4052 | 53.6 |  | 23 | 29.5 |  | 123 | 53.0 |  | **2.1x10^-5^** | ns |
| HLA-B37 | 270 | 3.6 |  | 13 | 16.7 |  | 10 | 4.4 |  | **1.1x10^-9^** | ns |
| HLA-DR2 | 1967 | 30.0 |  | 25 | 32.1 |  | 92 | 39.8 |  | ns | *1.4x10^-3^* |
| HLA-DR4 | 1969 | 30.0 |  | 23 | 29.5 |  | 43 | 18.6 |  | ns | **1.9x10^-4^** |
| HLA-DR5 | 1158 | 17.7 |  | 8 | 10.3 |  | 60 | 26.0 |  | ns | *1.2x10^-3^* |
| HLA-DR7 | 1246 | 19.0 |  | 7 | 9.0 |  | 25 | 10.8 |  | ns | *1.7x10^-3^* |
| HLA-DR10 | 118 | 1.8 |  | 6 | 7.7 |  | 5 | 2.2 |  | **1.3x10^-4^** | ns |

*****Significant differences (p<0.001) are shown in bold, suggestive ones (p<0.003) in italic. ns = non significant
