# Supplementary material for: HLA Associations in Classical Hodgkin Lymphoma: EBV Status Matters
Source: PLoS One. 2012 Jul 10;7(7):e39986. doi: 10.1371/journal.pone.0039986 (PMC3393726; doi:10.1371/journal.pone.0039986)
Supplement: Table S4 — Phenotype frequencies of HLA alleles with (nearly) significant difference between EBV+ or EBV− cHL patients. (DOC) [file pone.0039986.s005.doc]

**Supplementary Table S4.** Phenotype frequencies of HLA alleles with (nearly) significant difference between EBV+ or EBV- cHL patients

| **Allele** | **EBV+ cHL** | |  | **EBV- cHL** | |  | **p-value** |
| --- | --- | --- | --- | --- | --- | --- | --- |
|  | n | % |  | n | % |  |  |
| HLA-A1 | 43 | 55.1% |  | 70 | 30.2% |  | **7.4x10^-5^** |
| HLA-A2 | 23 | 29.5% |  | 123 | 53.0% |  | **3.2x10^‑4^** |
| HLA-B37 | 13 | 16.7% |  | 10 | 4.4% |  | **3.7x10^-4^** |
| HLA-Cw6 | 20 | 26.0% |  | 26 | 11.5% |  | *2.1x10^-3^* |

*****Significant differences (p<0.001) are shown in bold, suggestive ones (p<0.003) in italic.
